# Functional and Radiological Outcomes of Medial Malleolus Fracture Fixation Using Headless Compression Screws

**DOI:** 10.7759/cureus.83869

**Published:** 2025-05-10

**Authors:** Nikhilesh Das, Suman S Mishra, Anuraag Mohanty, Dhananjay Sahoo

**Affiliations:** 1 Department of Orthopaedics, Peerless Hospital and B.K. Roy Research Center, Kolkata, IND; 2 Department of Orthopaedics, Kalinga Institute of Medical Sciences, Bhubaneswar, IND; 3 Department of Orthopaedics, Apollo Hospitals, Bhubaneswar, IND

**Keywords:** ankle fractures, baird and jackson score, headless compression screw fixation, medial malleolar fractures, radiological outcomes

## Abstract

Introduction: Medial malleolar fractures frequently accompany ankle fractures and demand precise anatomical reduction for optimal outcomes. Several fixation techniques have been employed, including partially threaded unicortical lag screws, which, despite their ease of use, are associated with reduced torque resistance and increased screw loosening. Alternatively, partially threaded cancellous screws with or without washers have also been utilized to achieve stable fixation in medial malleolar injuries.

Objective: This study aimed to evaluate and compare the radiological and functional outcomes of medial malleolar fractures in ankle fracture cases managed using headless compression screw fixation.

Subjects and methods: A total of 47 patients were included in this retrospective study conducted over a 30-month period. Preoperative assessments included detailed clinical histories, injury mechanism analysis, and imaging investigations. Postoperative follow-up extended to 30 months. Clinical outcomes were assessed using the Baird and Jackson Scoring System (BJS) and the American Orthopaedic Foot and Ankle Society (AOFAS) Hindfoot Score.

Results: Among the 47 patients, 37 patients (78.7%) achieved AOFAS scores above 90, reflecting excellent functional outcomes, while 10 patients (21.3%) scored between 80 and 90, indicating good results. Additionally, 18 patients (38.2%) attained BJS scores greater than 95, corresponding to excellent outcomes in terms of pain relief, mobility, and radiological union.

Conclusion: Headless cannulated compression screw fixation demonstrates high efficacy in the treatment of medial malleolar fractures, offering a reliable alternative to conventional fixation techniques with favourable radiological and functional outcomes.

## Introduction

Medial malleolar fractures are among the most frequent injuries encountered in orthopaedic trauma settings. They are typically observed in association with ankle fractures, which together represent approximately 9% of all skeletal fractures globally [1]. The medial malleolus forms the distal aspect of the tibia and constitutes an essential component of the ankle mortise, playing a vital role in stabilizing the tibiotalar articulation. Given the intra-articular nature of medial malleolar fractures, they present a significant risk of compromising joint congruity [2]. Without accurate anatomical reduction and appropriate stabilization, these injuries can lead to persistent pain, mechanical instability, and the early onset of post-traumatic osteoarthritis [3]. Therefore, meticulous surgical management is considered the cornerstone of effective treatment to ensure the long-term functional recovery and prevention of degenerative changes.

Over the years, multiple surgical techniques have been introduced to address variations in fracture morphology, bone quality, and patient-specific factors. Commonly utilized fixation methods include partially threaded unicortical lag screws, partially threaded cancellous screws with or without washers, and perpendicular insertion of cortical or cancellous lag screws to maximize interfragmentary compression. Each of these options aims to achieve stable fixation by reducing micromotion at the fracture site and promoting osteogenesis during the early stages of healing [4]. Furthermore, when fractures demonstrate vertical shear components or comminution, the use of anti-glide plating has been advocated due to its superior biomechanical profile and resistance to vertical displacement forces [5]. Additionally, in cases involving small or osteoporotic fragments that are not amenable to screw fixation, suture anchor-based techniques have gained popularity for their ability to minimize hardware-related complications while ensuring adequate fragment stabilization [6,7].

From a biomechanical perspective, partially threaded unicortical lag screws, though commonly used, have demonstrated lower resistance to torque and have been associated with an increased incidence of screw loosening and non-union, particularly in osteoporotic bone or in fractures with significant vertical orientation [8]. In contrast, cancellous screws placed perpendicular to the fracture line have shown superior load distribution and interfragmentary compression, particularly in vertical shear fractures, thereby facilitating improved outcomes in terms of union and early mobilization [9]. Anti-glide plate constructs, while providing stiffer initial fixation and better load-bearing capacity, may be associated with soft tissue irritation, wound complications, or the need for hardware removal due to prominence over the subcutaneous border of the medial malleolus [10]. Suture anchors, meanwhile, represent a viable alternative in certain contexts; they eliminate the bulkiness of traditional hardware and are especially useful in patients with limited bone stock or in comminuted fractures where conventional fixation might exacerbate fragment displacement [11].

Despite the wealth of techniques available, the orthopaedic community continues to debate the most effective modality for medial malleolar fracture fixation. Recently, headless compression screws have emerged as a potentially superior alternative due to their ability to achieve interfragmentary compression while avoiding protrusion of hardware above the bone surface. These screws, by virtue of their design, reduce the risk of postoperative soft tissue irritation, minimize the necessity for secondary hardware removal, and theoretically improve patient comfort and recovery kinetics. However, despite these theoretical advantages, there remains a paucity of robust clinical evidence directly comparing the outcomes of headless compression screws with those of traditional fixation methods [12]. As such, the current study was undertaken with the objective of evaluating the functional and radiological outcomes in patients who underwent open reduction and internal fixation of medial malleolar fractures using headless compression screws. This study aims to address existing gaps in the literature and provide further insights into the efficacy, safety, and long-term performance of this evolving surgical technique.

## Materials and methods

This retrospective observational study was conducted in the Department of Orthopaedics at Peerless Hospital and B.K. Roy Research Center, Kolkata, West Bengal, India, over a 30-month duration from September 2018 to March 2021. Ethical clearance was obtained from the Peerless Hospitex Hospital and Research Center Limited Clinical Research Ethics Committee (approval number: PHH&RCL-CREC/S08/2021) before the initiation of the study, ensuring adherence to national guidelines for clinical research involving human subjects. Using a hospital-based electronic medical record system (TATA HMS), patients were screened based on outpatient and inpatient registrations. Inclusion criteria comprised patients aged 18 years and above, diagnosed with transverse, oblique, or vertical fractures of the medial malleolus, who underwent open reduction and internal fixation with headless cannulated compression screws and had functionally competent ipsilateral knee and contralateral lower limbs. This approach was intended to reduce variability in rehabilitation potential due to confounding factors such as bilateral lower limb dysfunction. Patients were excluded if they had bilateral lower limb injuries, polytrauma, open ankle fractures, significant ipsilateral tibial bone loss, or a history of prior medial malleolus fracture or lacked at least 12 months of postoperative data. From the available medical records, 47 patients who met the inclusion criteria were identified and included in the study. As this was a retrospective analysis, no direct contact with patients was required, and informed consent was waived or obtained as per institutional policy. Preoperative clinical data were extracted from patient charts, focusing on the mechanism of injury, foot positioning at the time of trauma, energy involved, and any prior treatment. Medical history, including comorbidities such as diabetes or peripheral vascular disease, was documented to assess potential influences on fracture healing.

Preoperative evaluation included detailed clinical history, mode of injury, energy involved, foot positioning at trauma, and comorbidities such as diabetes and peripheral vascular disease that could influence healing outcomes. Pre-surgical laboratory workup consisted of complete hemogram, renal function tests, electrolytes, coagulation profile, viral serology, and random blood glucose. Standard radiographic workup involved anteroposterior and lateral views of the ankle; where necessary, CT scans with three-dimensional reconstructions were used to evaluate complex fracture morphologies and assist in operative planning.

The Lauge-Hansen classification was used to stratify ankle fractures based on injury mechanism: Pronation-abduction (PA) involves medial tension failure with lateral compression injury. Pronation-external rotation (PER) begins with deltoid ligament or medial malleolus rupture, followed by fibular and syndesmotic disruption. Supination-adduction (SA) starts with lateral ligament damage or avulsion, ending with a vertical medial fracture. Supination-external rotation (SER) is the most common type, typically featuring fibular spiral fracture and medial malleolar disruption.

The Herscovici classification was applied to categorize medial malleolus fractures by fracture morphology as follows: Type A, avulsion of the tip of the medial malleolus; Type B, between the tip and intercollicular groove; Type C, at the intercollicular groove; and Type D, extending above the groove into the metaphysis.

**Figure 1:**
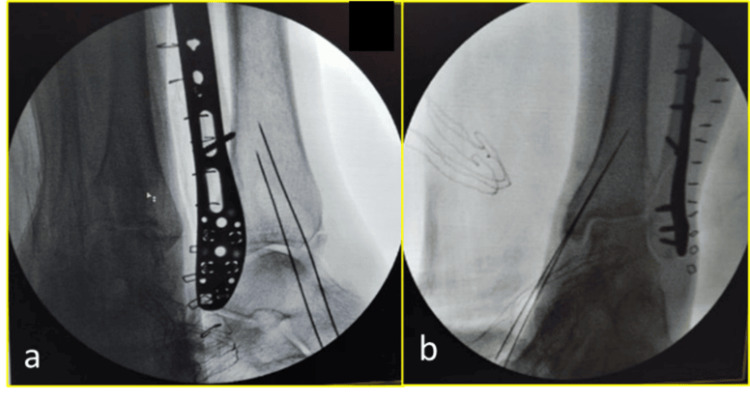
Intraoperative fluoroscopic view showing provisional fixation with K-wires before the placement of headless compression screws: (a) lateral view and (b) anteroposterior view The figure demonstrates alignment verification under image intensification during reduction.

**Figure 2:**
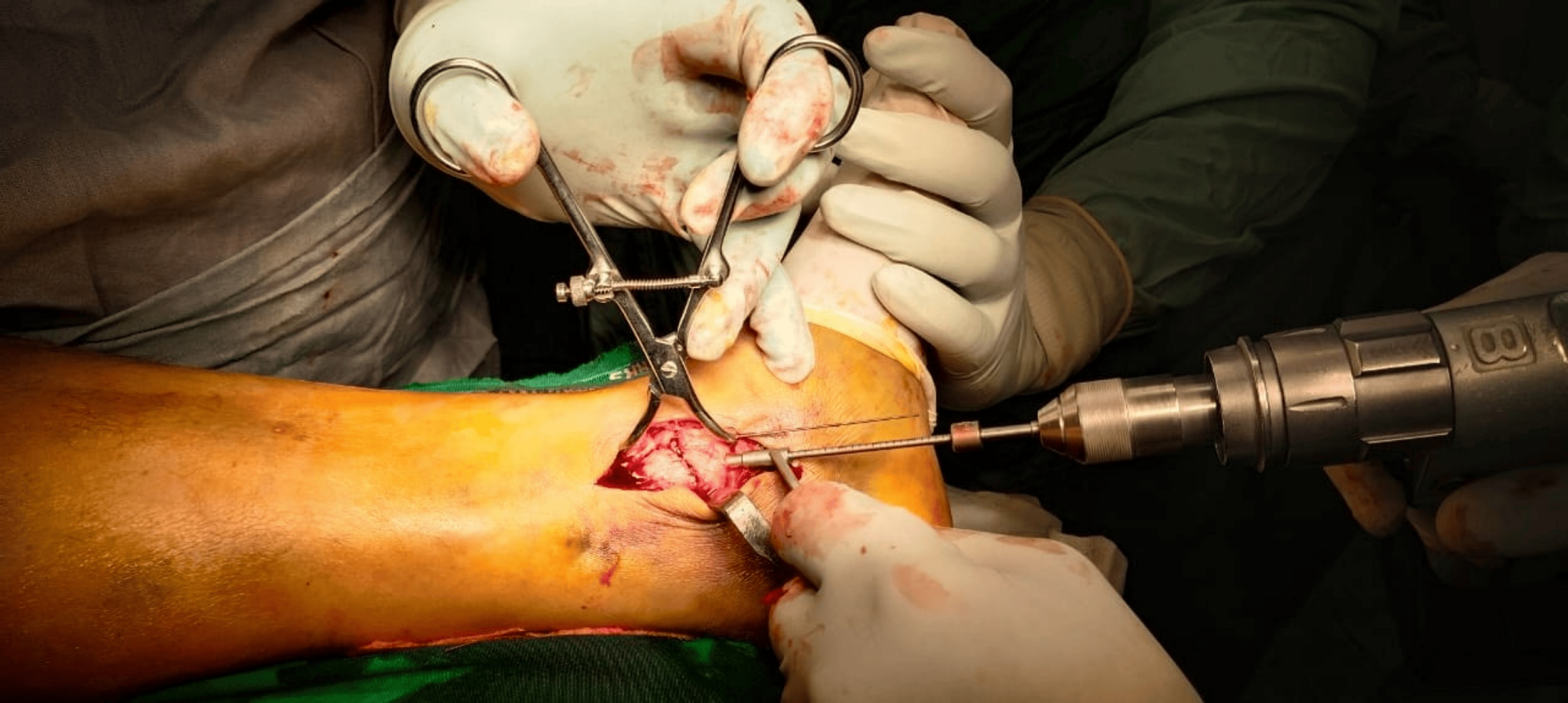
Clinical intraoperative image showing reduction forceps applied during drilling for headless screw insertion The figure illustrates the surgical technique used to maintain fragment stability during fixation.

All patients underwent pre-anesthesia check-ups, and surgeries were performed under either spinal or general anesthesia, depending on patient-specific factors. Temporary stabilization was achieved intraoperatively using K-wires (1.6 mm), followed by definitive fixation with headless cannulated compression screws under fluoroscopic images in Figure 1. Operative details such as the number and configuration of screws, surgical approach, and intraoperative complications were recorded. An intraoperative clinical image depicting the use of reduction forceps during drilling for screw insertion is shown Figure 2, highlighting the precision involved in screw placement. Postoperative radiographs were reviewed to confirm the quality of reduction and hardware positioning.

Patients were followed at two, four, six, 10, 14, and 18 weeks, with further evaluations at six, nine, 12, 24, and 30 months. Functional assessment at each follow-up included evaluation of pain, ankle mobility, gait, weight-bearing ability, and any complications. Clinical outcomes were evaluated using two validated scoring systems. Early postoperative recovery was assessed using the Baird and Jackson Scoring System (BJS) (Appendix 1), which considers five components, namely, pain (15 points), gait (10 points), range of motion (10 points), joint stability (15 points), and radiographic findings and return to activity (20 points), yielding a total score of 70 [13]. Outcomes were interpreted as excellent (≥95), good (90-94), fair (80-89), and poor (<80) [14]. Long-term outcomes were evaluated using the American Orthopaedic Foot and Ankle Society (AOFAS) Hindfoot Score (Appendix 2), which assigns 40 points to pain, 50 points to function (including walking distance, gait abnormalities, and joint mobility), and 10 points to alignment, totalling 100 points. These were interpreted as excellent (≥90), good (80-89), fair (70-79), and poor (<70) [15]. Both scoring systems have been previously validated and shown to demonstrate strong interobserver reliability in assessing ankle trauma outcomes.

Statistical analysis

The data collected were first compiled and organized using Microsoft Excel (Microsoft Corporation, Redmond, Washington, United States) for initial tabulation and sorting. Final statistical analysis was conducted using IBM SPSS Statistics for Windows, Version 22.0 (Released 2013; IBM Corp., Armonk, New York, United States). Descriptive statistics were applied to summarize demographic characteristics and clinical parameters. Continuous variables, such as age, functional scores, and follow-up duration, were expressed as means with standard deviations, while categorical variables, including sex, side of injury, mode of injury, and fracture classification types, were represented as frequencies and percentages. To compare early and late functional outcomes measured by BJS and AOFAS scores across different subgroups, such as age categories, fracture types, and injury mechanisms, the independent samples t-test was used for two-group comparisons, while the one-way analysis of variance (ANOVA) was employed for comparisons involving more than two groups. The chi-squared test was used to evaluate associations between categorical variables. A p-value of less than 0.05 was considered statistically significant for all comparative analyses.

## Results

**Figure 3:**
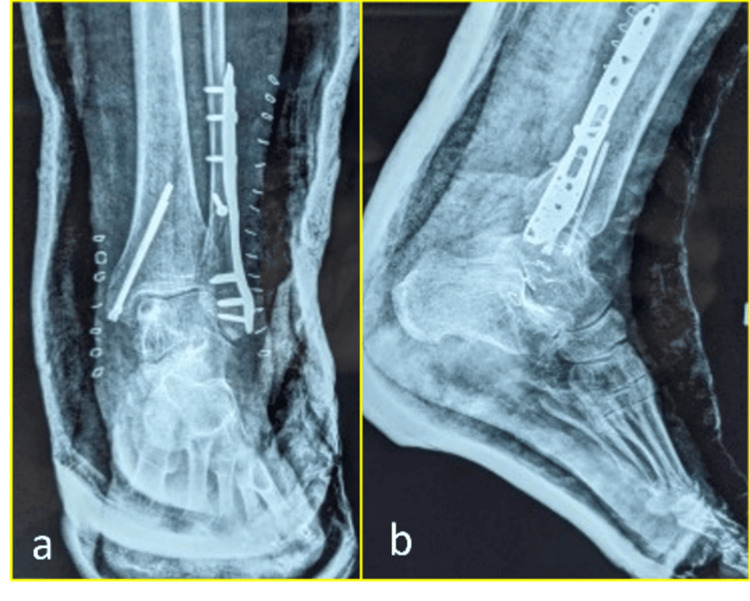
Postoperative anteroposterior and lateral radiographs confirming anatomical reduction and screw placement in a bimalleolar fracture: (a) anteroposterior view and (b) lateral view The lateral plate for fibular fixation and headless screw for the medial malleolus are visible.

A total of 47 patients were included in the study, all of whom underwent open reduction and internal fixation of medial malleolar fractures using headless cannulated compression screws. Of these, 31 (66%) were male and 16 (34%) were female. The mean age at the time of surgery was 39.94±9.15 years, with the age range spanning from 21 to 70 years. Postoperative radiographs confirmed anatomical reduction and stable fixation in all cases. In patients presenting with associated bimalleolar fractures (n=12), the lateral malleolus was fixed using a lateral plate in conjunction with the headless screw technique for the medial fragment (Figure 3).

Age had a statistically significant impact on clinical outcome, with patients aged below 40 years showing a higher frequency of excellent or good outcomes, as measured by both the AOFAS and BJS. The p-value was <0.001, indicating a strong correlation between younger age and improved postoperative functional scores. Conversely, gender showed no statistically significant influence on the outcome scores (p=0.716), suggesting that sex-related biomechanical differences did not impact the success of this fixation method.

The mode of injury was another significant variable influencing outcomes. Among the various mechanisms observed, road traffic accidents (n=22) were the most common, followed by low-energy twisting injuries (n=18) and falls from height (n=7). Patients sustaining injuries from high-energy trauma, such as road traffic accidents, had a greater proportion of excellent functional scores compared to those with low-energy mechanisms. The correlation between the mechanism of injury and final outcome scores was statistically significant, with a p-value of 0.008. These results suggest that while high-energy trauma can cause more complex injuries, aggressive early surgical intervention with stable fixation may facilitate better functional recovery. Data are summarized in Table 1 (AOFAS) and Table 2 (BJS).

**Table 1:** Mode of injury calculated through the AOFAS score The table shows the correlation between different trauma mechanisms (e.g., road traffic accident, twisting injury, fall) and the final AOFAS outcome categories (excellent, good, fair, poor). Statistical analysis was performed using the chi-squared test (p=0.008). AOFAS: American Orthopaedic Foot and Ankle Society

		Final interpretation and result		Total n (%)	P-value
		Good n (%)	Excellent n (%)		
Mode of injury	Fall from height	6 (60)	8 (21.62)	14 (29.79)	0.008
	Road traffic accident	2 (20)	23 (62.16)	25 (53.19)	
	Slip	2 (20)	1 (2.7)	3 (6.38)	
	Sports	0 (0)	5 (13.51)	5 (10.64)	
Total		10 (100)	37 (100)	47 (100)	-

**Table 2:** Mode of injury calculated through the BJS The table displays the distribution of BJS outcomes by injury mechanism. The chi-squared test showed significant association (p=0.008). BJS: Baird and Jackson Scoring System

		Final interpretation and result		Total n (%)	P-value
		Good n (%)	Excellent n (%)		
Mode of injury	Fall from height	6 (60)	8 (21.62)	14 (29.79)	0.008
	Road traffic accident	2 (20)	23 (62.16)	25 (53.19)	
	Slip	2 (20)	1 (2.7)	3 (6.38)	
	Sports	0 (0)	5 (13.51)	5 (10.64)	
Total		10 (100)	37 (100)	47 (100)	-

Regarding the laterality of injury, 27 patients had right-sided and 20 had left-sided medial malleolar fractures. The side of injury demonstrated a statistically significant association with final outcomes (p=0.030), with right-sided fractures demonstrating slightly better recovery profiles. The reason for this variation is unclear but may relate to differences in limb dominance and rehabilitation compliance.

The Lauge-Hansen classification was used to stratify ankle fractures based on injury mechanism. SER injuries were the most prevalent (n=30), followed by SA (n=8), PA (n=5), and PER (n=4). Despite this distribution, the classification did not show a statistically significant association with functional outcomes (p=0.667), as detailed in Table 3 and Table 4. This finding implies that the fracture mechanism, though useful for understanding pathoanatomy, may not independently predict long-term recovery if fixation is appropriately performed.

**Table 3:** Lauge-Hansen classification according to the AOFAS score The table compares the functional outcomes according to fracture mechanism type. No statistically significant association was found (p=0.667). AOFAS: American Orthopaedic Foot and Ankle Society; PA: pronation-abduction; PER: pronation-external rotation; SA: supination-adduction; SER: supination-external rotation

		Final interpretation and result		Total n (%)	P-value
		Good n (%)	Excellent n (%)		
Lauge-Hansen classification	PA	1 (10)	2 (5.41)	3 (6.38)	0.667
	PER	4 (40)	10 (27.03)	14 (29.79)	
	SA	1 (10)	4 (10.81)	5 (10.64)	
	SER	4 (40)	21 (56.76)	25 (53.19)	
Total		10 (100)	37 (100)	47 (100)	-

**Table 4:** Lauge-Hansen classification according to the BJS The table compares the BJS-based functional outcomes across different Lauge-Hansen classification types. Chi-squared statistical test: p=0.667. BJS: Baird and Jackson Scoring System; PA: pronation-abduction; PER: pronation-external rotation; SA: supination-adduction; SER: supination-external rotation

		Final interpretation and result		Total n (%)	P-value
		Good n (%)	Excellent n (%)		
Lauge-Hansen classification	PA	1 (10)	2 (5.41)	3 (6.38)	0.667
	PER	4 (40)	10 (27.03)	14 (29.79)	
	SA	1 (10)	4 (10.81)	5 (10.64)	
	SER	4 (40)	21 (56.76)	25 (53.19)	
Total		10 (100)	37 (100)	47 (100)	-

The Herscovici classification was applied to categorize medial malleolus fractures by fracture morphology. Type B (n=21) and Type C (n=17) were the most frequent, followed by Type A (n=5) and Type D (n=4). There was no statistically significant difference in outcomes between the various Herscovici types (p=0.187), as shown in Table 5 and Table 6.

**Table 5:** Herscovici classification according to the AOFAS score The table shows the functional outcomes stratified by fracture morphology using AOFAS scoring. No significant association was found (p=0.187). AOFAS: American Orthopaedic Foot and Ankle Society; Type B: fracture between the tip and intercollicular groove; Type C: at the level of the intercollicular groove; Type D: extending above the intercollicular groove into the metaphysis

		Final interpretation and result		Total n (%)	P-value
		Good n (%)	Excellent n (%)		
Herscovici classification	B	2 (20)	19 (51.35)	21 (44.68)	0.187
	C	7 (70)	14 (37.84)	21 (44.68)	
	D	1 (10)	4 (10.81)	5 (10.64)	
Total		10 (100)	37 (100)	47 (100)	-

**Table 6:** Herscovici classification according to the BJS The table compares the BJS score distributions among fracture types defined by the Herscovici system. Chi-squared test result: p=0.187. BJS: Baird and Jackson Scoring System; Type B: fracture between the tip and intercollicular groove; Type C: at the level of the intercollicular groove; Type D: extending above the intercollicular groove into the metaphysis

		Final interpretation and result		Total n (%)	P-value
		Good n (%)	Excellent n (%)		
Herscovici classification	B	2 (20)	19 (51.35)	21 (44.68)	0.187
	C	7 (70)	14 (37.84)	21 (44.68)	
	D	1 (10)	4 (10.81)	5 (10.64)	
Total		10 (100)	37 (100)	47 (100)	-

Functional outcomes (AOFAS)

Out of 47 patients, 38 achieved the maximum pain sub-score of 40 (no pain), while nine patients scored 30, indicating mild, occasional discomfort. Regarding activity limitations, 32 patients scored 7, indicating minimal restrictions, and 15 scored 10, denoting full activity. For the walking-distance item, 41 patients (87.2%) achieved the maximum score of 5 (>6 blocks≈480 m), whereas six patients (12.8%) scored 4 (<6 blocks). For the gait-abnormality item, 43 patients (91.5%) scored 8 or 9 (no/slight abnormality), and four patients (8.5%) scored 4 (obvious abnormality). These values are reported as individual AOFAS sub-scores and should not be interpreted as a formal gait analysis. Sagittal mobility was preserved in 45 patients (score: 8), and inversion/eversion was >75% of normal in 44 patients (score: 6). Ankle-hindfoot stability was maintained in all patients (score: 8), and alignment was anatomically correct in 46 out of 47 patients (score: 10). Overall, 37 patients scored ≥90 (excellent), while the remaining 10 scored 80-89 (good).

Functional outcomes (BJS)

Pain scores of 15 were recorded in 39 patients, indicating complete pain relief. Eight patients reported mild pain (score: 12). Ankle stability was excellent in 44 patients (score: 15), with three reporting instability during sports activities (score: 5). Ambulatory function was optimal in 43 patients (score: 15), while four had slight restrictions (score: 12). None of the patients could run pain-free; however, 22 managed with slight discomfort (score: 8), and 25 reported moderate limitations (score: 6). Range of motion was graded excellent in 20 patients (score: 10), good in 27 (score: 7), and moderate in one (score: 4). Regarding return to work, 44 patients resumed their usual occupation with minimal adjustment (score: 8), and three required significant modification (score: 6). Radiologically, 44 patients maintained a preserved ankle mortise with no signs of degenerative changes; three patients exhibited minor reactive sclerosis during the one-year follow-up.

The final BJS score distribution is as follows: excellent (≥95): 18 patients; good (90-94): 17 patients; fair (80-89): eight patients; and poor (<80): four patients.

**Figure 4:**
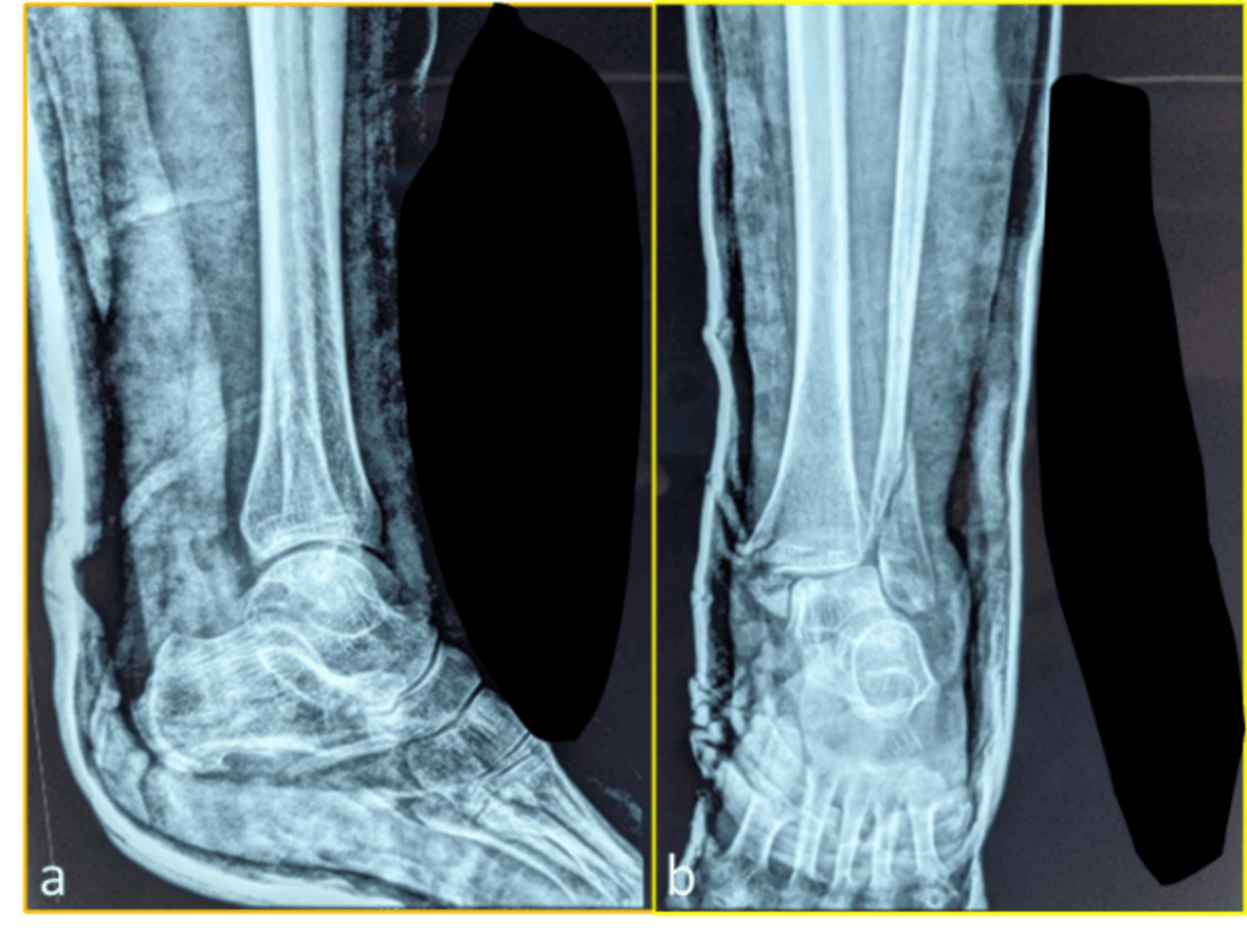
Postoperative photograph at the six-month follow-up showing well-healed medial incision without signs of infection or hypertrophic scarring: (a) lateral view and (b) anteroposterior view

**Figure 5:**
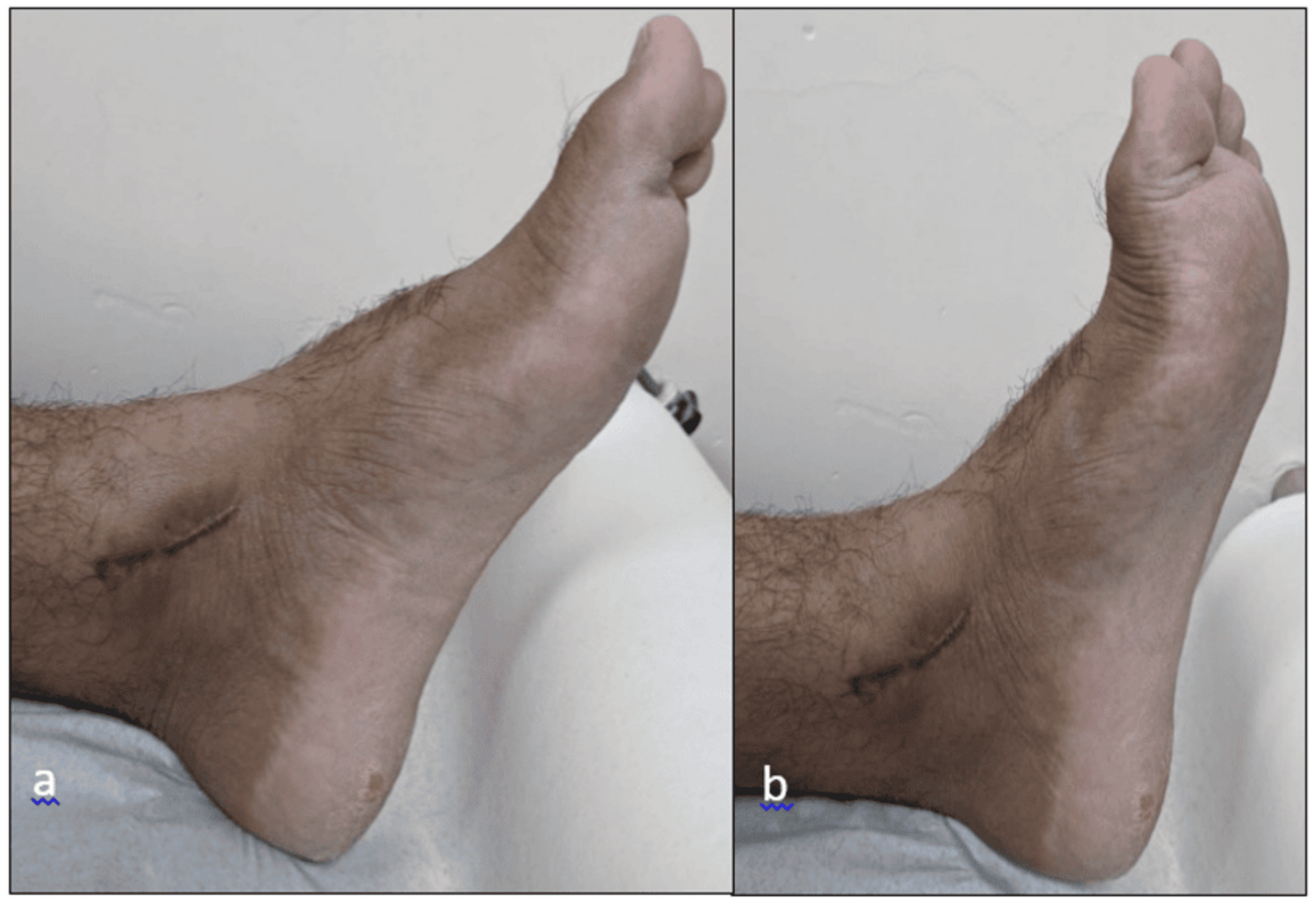
Photograph at the final follow-up demonstrating satisfactory cosmetic outcome and absence of postoperative complications

Postoperative healing was uneventful in all cases. There were no incidences of wound infection, implant failure, or deep vein thrombosis. Scar healing was satisfactory and aesthetically acceptable, with representative cases shown in Figure 4 and Figure 5. These results suggest that medial malleolus fixation with headless cannulated screws leads to consistently favourable outcomes when combined with standardized surgical protocols and structured rehabilitation.

## Discussion

The primary objective in the surgical management of medial malleolar fractures is to achieve stable internal fixation that permits early mobilization, effective rehabilitation, and reliable anatomical restoration. Several fixation techniques have been documented in the literature, including K-wire and tension band wiring, partially threaded lag screws, suture anchors, bioabsorbable implants, and, more recently, headless cannulated compression screws. While K-wires and tension band constructs are technically simple, they are frequently associated with complications such as soft tissue irritation, pin migration, and hardware prominence that often necessitate secondary procedures [16]. Similarly, although bioabsorbable screws eliminate the need for hardware removal, they may lead to foreign body reactions in some patients [17]. Suture anchors, although advantageous in cases involving osteoporotic or comminuted fragments, are limited by their cost, technical complexity, and learning curve [18].

In this context, headless cannulated compression screws offer a promising alternative by providing stable fixation without the prominence of screw heads that may irritate the overlying soft tissue. Their subcortical design reduces the likelihood of implant-related symptoms and facilitates early joint motion, which is crucial in preventing stiffness and preserving long-term function [19]. The current study supports these assertions, as 85% of patients achieved excellent outcomes based on validated scoring systems, and radiographic assessments confirmed union in over 90% of cases within the expected healing timeframe. These results are in alignment with existing biomechanical evidence that headless screws provide superior interfragmentary compression while avoiding stress shielding, thereby optimizing conditions for bone healing [18].

The findings of this investigation are consistent with previously published clinical studies. Tekin et al. demonstrated favourable results using anterograde headless cannulated screws in Herscovici Type B fractures, with no requirement for implant removal or revision. Their study highlighted not only radiological success but also favourable cosmetic and functional outcomes. Similarly, Bulut and Gursoy conducted a comparative study in which headless screws resulted in significantly reduced pain on palpation and a lower incidence of implant-related discomfort compared to conventional cancellous screws. Although time to union was comparable between groups, patient satisfaction and postoperative mobility were notably improved in the headless screw cohort [20].

Additional evidence from larger cohort studies also reinforces these observations. Kochai et al. [21] reported that patients treated with fully threaded headless screws experienced faster union and fewer instances of soft tissue irritation compared to those managed with tension band wiring or partially threaded lag screws. Their results echo the current findings, particularly in terms of implant tolerance, range of motion preservation, and early return to functional activities. The mechanical advantage of headless screws lies in their ability to deliver controlled compression across the fracture site while avoiding the mechanical prominence associated with traditional screw heads, thereby reducing local irritation and enhancing patient compliance during rehabilitation [3].

Despite these encouraging outcomes, this study has several limitations. Firstly, its retrospective observational design may introduce selection and reporting bias, particularly regarding follow-up compliance and subjective outcome reporting. Secondly, the sample size of 47 patients, while adequate for descriptive analysis, limits statistical power when performing subgroup comparisons, especially in variables such as fracture classification and mechanism of injury. Thirdly, the study did not include a comparative group treated with traditional fixation methods, thereby restricting the ability to draw definitive conclusions regarding the superiority of headless screws. Additionally, long-term complications such as post-traumatic osteoarthritis or late hardware intolerance were not assessed beyond the 30-month follow-up period. Lastly, although validated scoring systems were employed, functional outcomes could still be influenced by patient-specific factors, rehabilitation variability, and personal pain thresholds, which were not standardized in this review.

## Conclusions

The findings of this retrospective study provide compelling evidence in favour of headless cannulated compression screw fixation as an effective surgical approach for the management of medial malleolar fractures. The technique demonstrated consistently favourable outcomes across radiological and functional parameters, with all fractures in the study cohort achieving union within the expected healing period. A significant majority of patients reported excellent postoperative function, as measured by both the AOFAS Hindfoot Score and BJS, reflecting a high level of ankle joint stability, mobility, and pain relief. These functional gains were supported by radiographic evidence of anatomical alignment and preserved joint spaces at follow-up. Importantly, no patients in the series experienced postoperative complications such as non-union, wound infection, or hardware failure. The absence of secondary surgical procedures for implant removal further underscores the technique's advantage in minimizing hardware-related morbidity. This can be attributed to the biomechanical design of the headless screw, which allows for interfragmentary compression without protruding hardware, thereby reducing the risk of soft tissue irritation, a common limitation of traditional implants.

In addition to its clinical effectiveness, the method proved suitable across various fracture morphologies and injury mechanisms, showing consistent outcomes regardless of Lauge-Hansen or Herscovici classification types. Although no statistically significant outcome variation was observed among different fracture patterns, the technique provided stable fixation and high functional recovery in both low-energy and high-energy injury scenarios. These findings suggest a degree of versatility that supports broader clinical adoption. From a surgical standpoint, the headless screw technique is straightforward to execute with proper training and can be integrated into standard fixation protocols without requiring specialized instrumentation or implants beyond those commonly available. Its capacity to deliver stable, subcortical fixation while preserving soft tissue integrity makes it particularly advantageous in anatomically sensitive areas such as the medial malleolus.

Taken together, the results of this study affirm the clinical utility of headless cannulated compression screws in achieving durable, complication-free outcomes in medial malleolar fracture fixation. While the retrospective nature and relatively limited sample size of the study restrict broader generalization, the consistently positive results across all outcome measures provide a strong foundation for recommending this technique as a preferred option in appropriately selected patients. Larger prospective studies with extended follow-up will be valuable in corroborating these findings and in determining long-term joint preservation and functional outcomes. Nonetheless, the current evidence supports the integration of headless compression screw fixation as a reliable and potentially superior method in the operative management of medial malleolar fractures.

## Appendices

Appendix 1

**Table 7:** Baird and Jackson master chart BJS score interpretation: excellent: ≥95; good: 90-94; fair: 80-89; poor: <80 BJS: Baird and Jackson Scoring System; RTA: road traffic accident; POP: Plaster of Paris (immobilization); SUPF INF: superficial infection; STIFF: joint stiffness (post-op complication); PA: pronation-abduction; PER: pronation-external rotation; SA: supination-adduction; SER: supination-external rotation; Type B: fracture between the tip and intercollicular groove; Type C: at the intercollicular groove; Type D: extending above the intercollicular groove into the metaphysis; NIL: no complication

Serial number	Name	Age	Gender	Mode of injury	Side of injury	Lauge-Hansen classification	Herscovici classification	Time interval between injury and surgery (days)	Complication	Time of immobilization by POP slab (days)	Time of full weight-bearing exercises (days)	Duration of follow-up (in months)	BJS								
													Pain (15)	Stability of the ankle (15)	Able to walk (15)	Able to run (10)	Ability to work (10)	The motion of the ankle (10)	Radiographic result (25)	Total	Final interpretation and result
1	AB	41	M	RTA	R	SER	C	5	NIL	30	90	24	15	15	15	8	8	10	25	96	Excellent
2	SN	26	M	Sports	R	SA	D	1	NIL	30	42	24	15	15	15	8	8	10	25	96	Excellent
3	MP	40	F	RTA	L	SER	B	3	NIL	30	42	24	15	15	15	6	8	7	15	81	Fair
4	SR	51	M	Fall from height	R	SER	B	5	STIFF	30	90	30	12	5	15	6	8	7	25	78	Poor
5	SG	54	M	Fall from height	L	PER	C	6	SUPF INF	30	90	30	15	15	12	6	6	4	15	73	Poor
6	GD	25	F	Sports	R	PA	C	2	NIL	30	90	24	15	15	15	8	8	10	25	96	Excellent
7	SS	40	M	RTA	L	PER	C	1	NIL	30	90	24	15	15	15	8	8	10	25	96	Excellent
8	RH	58	F	Slip	L	SA	D	3	NIL	30	42	24	15	15	15	8	8	10	25	96	Excellent
9	AB	43	M	RTA	R	PER	C	1	NIL	30	90	24	15	15	15	8	8	10	25	96	Excellent
10	TG	38	M	RTA	R	SER	B	1	NIL	30	90	30	15	15	15	6	8	7	25	91	Good
11	MD	55	M	Fall from height	L	PER	C	3	NIL	30	90	24	12	15	15	6	8	7	25	88	Fair
12	SI	28	M	Sports	R	SER	B	3	NIL	30	90	24	15	15	15	8	8	10	25	96	Excellent
13	GD	64	F	Slip	L	PER	C	6	NIL	30	90	24	15	15	15	8	8	10	25	96	Excellent
14	RND	44	M	Fall from height	R	SA	D	1	NIL	30	90	24	15	15	15	6	8	7	15	81	Fair
15	SM	30	F	RTA	L	SER	B	2	NIL	30	90	24	15	15	15	8	8	7	25	93	Good
16	SD	38	M	RTA	R	SA	D	2	NIL	30	90	24	15	15	15	6	8	7	25	91	Good
17	AB	45	F	Fall from height	R	SER	B	3	NIL	30	90	24	15	15	15	8	8	10	25	96	Excellent
18	TSG	30	M	RTA	L	SER	B	5	NIL	30	42	24	15	15	15	8	8	10	25	96	Excellent
19	JG	60	M	Slip	L	SER	C	2	STIFF	30	90	24	12	5	15	6	8	7	25	78	Poor
20	KB	42	F	RTA	L	PER	C	2	NIL	30	90	24	15	15	15	6	8	7	25	91	Good
21	BM	31	M	Sports	R	SER	B	1	NIL	30	90	24	15	15	15	6	8	7	25	91	Good
22	AB	39	M	RTA	L	SER	B	2	NIL	30	90	24	15	15	15	8	8	10	25	96	Excellent
23	JM	44	M	RTA	R	PA	C	3	STIFF	30	90	24	12	5	15	6	8	7	25	78	Poor
24	SP	32	M	RTA	R	SER	B	3	NIL	30	90	24	15	15	15	8	8	10	25	96	Excellent
25	BG	50	F	Fall from height	R	SER	C	5	NIL	30	90	24	15	15	15	8	8	7	25	93	Good
26	AC	36	M	RTA	R	SER	B	6	NIL	30	90	24	15	15	15	8	8	7	25	93	Good
27	SP	34	M	RTA	R	SA	D	1	NIL	30	42	24	15	15	15	6	8	10	25	94	Good
28	KB	35	M	RTA	L	SER	B	2	NIL	30	90	24	15	15	15	8	8	10	25	96	Excellent
29	SC	48	F	Fall from height	L	PER	C	2	STIFF	30	90	24	12	15	12	6	6	7	25	83	Fair
30	AB	36	M	RTA	L	SER	B	2	NIL	30	90	24	15	15	15	8	8	7	25	93	Good
31	DRC	49	M	Fall from height	R	SER	B	6	NIL	30	90	24	15	15	15	8	8	10	25	96	Excellent
32	BK	38	M	RTA	R	PER	C	3	NIL	30	90	24	15	15	15	8	8	7	25	93	Good
33	MC	43	F	Fall from height	L	SER	B	2	STIFF	30	90	24	12	15	12	6	8	7	25	85	Fair
34	RM	39	M	RTA	L	SER	B	3	NIL	30	90	24	15	15	15	6	8	7	25	91	Good
35	SN	45	M	Fall from height	R	PER	C	2	NIL	30	42	24	15	15	15	6	8	7	25	91	Good
36	DB	27	F	Sports	L	PA	B	3	NIL	30	90	24	15	15	12	6	6	7	25	86	Fair
37	AS	29	M	RTA	R	SER	C	2	NIL	30	90	24	15	15	15	8	8	10	25	96	Excellent
38	AM	29	M	RTA	L	SER	B	2	NIL	30	90	24	15	15	15	8	8	7	25	93	Good
39	AM	38	M	Fall from height	R	SER	B	2	NIL	30	90	30	15	15	15	6	8	7	25	91	Good
40	AM	37	F	RTA	R	PER	C	2	NIL	30	42	24	15	15	15	6	8	7	25	91	Good
41	AM	35	M	RTA	R	PER	C	2	NIL	30	90	24	15	15	15	8	8	10	25	96	Excellent
42	AM	39	F	Fall from height	L	SER	C	2	SUPF INF	30	90	24	12	15	15	6	8	7	25	88	Fair
43	AM	33	F	RTA	L	SER	B	2	NIL	30	90	24	15	15	15	8	8	10	25	96	Excellent
44	AM	31	M	RTA	L	SER	B	2	NIL	30	42	24	15	15	15	8	8	7	25	93	Good
45	AM	38	F	Fall from height	R	PER	C	2	NIL	30	90	30	15	15	15	8	8	10	25	96	Excellent
46	AM	43	M	RTA	L	PER	C	2	NIL	30	90	24	12	15	15	6	8	7	25	88	Fair
47	AM	47	F	Fall from height	R	PER	C	2	NIL	30	90	24	15	15	15	6	8	7	25	91	Good

Appendix 2

**Table 8:** AOFAS Hindfoot Score master chart Final AOFAS score interpretation: excellent: 90-100; good: 80-89; fair: 70-79; poor: <70 AOFAS: American Orthopaedic Foot and Ankle Society; RTA: road traffic accident; POP: Plaster of Paris (immobilization); SUPF INF: superficial infection; STIFF: joint stiffness (post-op complication); PA: pronation-abduction; PER: pronation-external rotation; SA: supination-adduction; SER: supination-external rotation; Type B: fracture between the tip and intercollicular groove; Type C: at the intercollicular groove; Type D: extending above the intercollicular groove into the metaphysis; NIL: no complication

Serial number	Age	Gender	Mode of injury	Side of injury	Lauge-Hansen classification	Herscovici classification	Time interval between injury and surgery (days)	Complication	Time of immobilization by POP slab (days)	Time of full weight-bearing exercises (days)	Duration of follow-up (in months)	AOFAS Hindfoot Score										
												Pain (40)	Function (50)									
													Activity limitation support requirement (10)	Maximum walking distance blocks (5) (1 block=80.4 meters)	Walking surface (5)	Gait abnormality (8)	Sagittal motion (8)	Hindfoot motion (6)	Ankle-hindfoot stability (8)	Alignment (10)	Total	Final interpretation and result
1	41	M	RTA	R	SER	C	5	NIL	30	90	24	40	7	5	3	8	8	6	8	10	95	Excellent
2	26	M	Sports	R	SA	D	1	NIL	30	42	24	40	10	5	3	8	8	6	8	10	98	Excellent
3	40	F	RTA	L	SER	B	3	NIL	30	42	24	40	7	5	3	8	8	3	8	10	92	Excellent
4	51	M	Fall from height	R	SER	B	5	STIFF	30	90	30	30	7	5	3	8	8	6	8	10	85	Good
5	54	M	Fall from height	L	PER	C	6	SUPF INF	30	90	30	40	10	4	3	4	4	3	8	10	86	Good
6	25	F	Sports	R	PA	C	2	NIL	30	90	24	40	10	5	3	8	8	6	8	10	98	Excellent
7	40	M	RTA	L	PER	C	1	NIL	30	90	24	40	7	5	5	8	8	6	8	10	97	Excellent
8	58	F	Slip	L	SA	D	3	NIL	30	42	24	30	10	4	3	4	8	6	8	10	83	Good
9	43	M	RTA	R	PER	C	1	NIL	30	90	24	40	7	5	5	8	8	6	8	10	97	Excellent
10	38	M	RTA	R	SER	B	1	NIL	30	90	30	40	7	5	3	8	8	6	8	10	95	Excellent
11	55	M	Fall from height	L	PER	C	3	NIL	30	90	24	30	7	5	3	8	8	6	8	10	85	Good
12	28	M	Sports	R	SER	B	3	NIL	30	90	24	40	10	5	5	8	8	6	8	10	100	Excellent
13	64	F	Slip	L	PER	C	6	NIL	30	90	24	40	10	5	3	8	8	6	8	10	98	Excellent
14	44	M	Fall from height	R	SA	D	1	NIL	30	90	24	40	7	5	3	8	8	3	8	10	92	Excellent
15	30	F	RTA	L	SER	B	2	NIL	30	90	24	40	7	5	5	8	8	6	8	10	97	Excellent
16	38	M	RTA	R	SA	D	2	NIL	30	90	24	40	7	5	3	8	8	6	8	10	95	Excellent
17	45	F	Fall from height	R	SER	B	3	NIL	30	90	24	40	10	5	3	8	8	6	8	10	98	Excellent
18	30	M	RTA	L	SER	B	5	NIL	30	42	24	40	10	5	3	8	8	6	8	10	99	Excellent
19	60	M	Slip	L	SER	C	2	STIFF	30	90	24	30	7	5	3	8	8	6	8	10	85	Good
20	42	F	RTA	L	PER	C	2	NIL	30	90	24	40	7	5	3	8	8	6	8	10	95	Excellent
21	31	M	Sports	R	SER	B	1	NIL	30	90	24	40	7	5	3	8	8	6	8	10	95	Excellent
22	39	M	RTA	L	SER	B	2	NIL	30	90	24	40	10	5	3	8	8	6	8	10	98	Excellent
23	44	M	RTA	R	PA	C	3	STIFF	30	90	24	30	7	5	3	8	8	6	8	10	85	Good
24	32	M	RTA	R	SER	B	3	NIL	30	90	24	40	7	5	3	8	8	6	8	10	95	Excellent
25	50	F	Fall from height	R	SER	C	5	NIL	30	90	24	40	7	5	3	8	8	6	8	8	93	Excellent
26	36	M	RTA	R	SER	B	6	NIL	30	90	24	40	7	5	3	8	8	6	8	10	95	Excellent
27	34	M	RTA	R	SA	D	1	NIL	30	42	24	40	7	5	3	8	8	6	8	10	95	Excellent
28	35	M	RTA	L	SER	B	2	NIL	30	90	24	40	10	5	3	8	8	6	8	10	98	Excellent
29	48	F	Fall from height	L	PER	C	2	STIFF	30	90	24	30	7	4	3	4	8	6	8	10	80	Good
30	36	M	RTA	L	SER	B	2	NIL	30	90	24	40	7	5	3	8	8	6	8	10	95	Excellent
31	49	M	Fall from height	R	SER	B	6	NIL	30	90	24	40	10	4	3	8	7	6	8	10	96	Excellent
32	38	M	RTA	R	PER	C	3	NIL	30	90	24	40	7	5	3	8	8	6	8	10	95	Excellent
33	43	F	Fall from height	L	SER	B	2	STIFF	30	90	24	30	7	4	3	8	8	6	8	10	84	Good
34	39	M	RTA	L	SER	B	3	NIL	30	90	24	40	7	5	3	8	8	6	8	10	95	Excellent
35	45	M	Fall from height	R	PER	C	2	NIL	30	42	24	40	7	5	3	8	8	6	8	10	95	Excellent
36	27	F	Sports	L	PA	B	3	NIL	30	90	24	40	7	4	3	4	8	6	8	10	90	Excellent
37	29	M	RTA	R	SER	C	2	NIL	30	90	24	40	10	5	3	8	8	6	8	10	98	Excellent
38	29	M	RTA	L	SER	B	2	NIL	30	90	24	40	7	5	3	8	8	6	8	10	95	Excellent
39	38	M	Fall from height	R	SER	B	2	NIL	30	90	30	40	7	5	3	8	8	6	8	10	95	Excellent
40	37	F	RTA	R	PER	C	2	NIL	30	42	24	40	7	5	3	8	8	6	8	10	95	Excellent
41	35	M	RTA	R	PER	C	2	NIL	30	90	24	40	10	5	3	8	8	6	8	10	98	Excellent
42	39	F	Fall from height	L	SER	C	2	SUPF INF	30	90	24	30	7	5	3	8	8	6	8	10	85	Good
43	33	F	RTA	L	SER	B	2	NIL	30	90	24	40	10	5	3	8	8	6	8	10	98	Excellent
44	31	M	RTA	L	SER	B	2	NIL	30	42	24	40	7	5	3	8	8	6	8	10	95	Excellent
45	38	F	Fall from height	R	PER	C	2	NIL	30	90	30	40	10	5	3	8	8	6	8	10	98	Excellent
46	43	M	RTA	L	PER	C	2	NIL	30	90	24	30	7	5	3	8	8	6	8	10	85	Good
47	47	F	Fall from height	R	PER	C	2	NIL	30	90	24	40	7	5	3	8	8	6	8	10	95	Excellent
